# Correction: Design of forced intercalation-induced light-up Rev peptide probes for HIV RRE RNA

**DOI:** 10.1039/d6ra90134d

**Published:** 2026-09-28

**Authors:** He Luo, Yusuke Sato, Seiichi Nishizawa

**Affiliations:** a Department of Chemistry, Graduate School of Science, Tohoku University 6-3 Aramaki-Aza Aoba, Aoba-ku Sendai 980-8578 Japan yusuke.sato.a7@tohoku.ac.jp seiichi.nishizawa.c8@tohoku.ac.jp; b JST, FOREST 4-1-8 Honcho, Kawaguchi Saitama 332-0012 Japan

## Abstract

Correction for ‘Design of forced intercalation-induced light-up Rev peptide probes for HIV RRE RNA’ by He Luo *et al.*, *RSC Adv.*, 2026, https://doi.org/10.1039/d6ra02799g.

The authors regret that the name of one of the authors, He Luo, was presented incorrectly in the original article. The corrected list of authors for this paper is as shown above.

The Royal Society of Chemistry apologises for these errors and any consequent inconvenience to authors and readers.

